# Entropy-Based Analysis and Bioinformatics-Inspired Integration of Global Economic Information Transfer

**DOI:** 10.1371/journal.pone.0051986

**Published:** 2013-01-02

**Authors:** Jinkyu Kim, Gunn Kim, Sungbae An, Young-Kyun Kwon, Sungroh Yoon

**Affiliations:** 1 Department of Electrical and Computer Engineering, Seoul National University, Seoul, Republic of Korea; 2 Department of Physics, Sejong University, Seoul, Republic of Korea; 3 School of Economics, Singapore Management University, Singapore, Singapore; 4 Department of Physics and Research Institute for Basic Sciences, Kyung Hee University, Seoul, Republic of Korea; University College of London - Institute of Neurology, United Kingdom

## Abstract

The assessment of information transfer in the global economic network helps to understand the current environment and the outlook of an economy. Most approaches on global networks extract information transfer based mainly on a single variable. This paper establishes an entirely new bioinformatics-inspired approach to integrating information transfer derived from multiple variables and develops an international economic network accordingly. In the proposed methodology, we first construct the transfer entropies (TEs) between various intra- and inter-country pairs of economic time series variables, test their significances, and then use a weighted sum approach to aggregate information captured in each TE. Through a simulation study, the new method is shown to deliver better information integration compared to existing integration methods in that it can be applied even when intra-country variables are correlated. Empirical investigation with the real world data reveals that Western countries are more influential in the global economic network and that Japan has become less influential following the Asian currency crisis.

## Introduction

Determining how information transfers in a global network is helpful in revealing the economic conditions of a country; it may also be a key to predicting future changes. However, the modern macroeconomy is too large and complex to build accurate models that can mimic the underlying economic system [1]. Although various approaches have shown considerable advances [2]–[5], major challenges must be dealt with for a complete understanding of the macroeconomy [4], [6]. One major challenge is the evaluation of direct or indirect interactions among agents (participants). We can consider an economic system as a complex network consisting of interacting market participants. Current economic models sometimes fail to predict emergent economic phenomena (*e.g.*, worldwide financial crises) from such a network.

Two goals of econophysics are to scrutinize the complex interactions between multiple agents in the economic system and to predict emergent economic phenomena. Various time-series analysis approaches have been introduced; and have achieved good progress by utilizing probability distribution [7]–[9], autocorrelation [10], multi-fractal approaches [11], [12], complexity [13], and transfer entropy [14] to analyze stock market indices.

Although network analysis using a single economic indicator has been useful, a large number of economic variables should be taken into account to understand the economic system from a holistic viewpoint. To gain insight into the structural properties of interaction networks, an integrative approach considering multiple economic variables is needed.

From other fields, we may learn how to incorporate information from multiple data sources. In biology, researchers construct gene networks showing the interactions between the genes of an organism [15]. These interactions are often too complex to assess within a single study. Therefore, multiple, independently curated gene-interaction databases for a single organism were created. To obtain a realistic picture of the full gene network, information scattered across different databases must be combined. For example, an integrative approach [16], [17] was employed to determine the yeast gene network, and produced a more accurate genetic interaction network than traditional approaches that had relied on a single data source. Rhodes *et al.* introduced a method that could integrate multiple data sources to obtain accurate protein-protein interactions [18]. Further on, we compare this method with that used in the present study.

In principle, we can utilize the integrative methods developed in biology to construct an economic network, but applying such methods directly to economic systems is difficult, given the many differences between the disciplines. In biology, each of the databases to be integrated often corresponds to a certain piece of a common puzzle. Biologists want to construct a gene network by patching together multiple databases that represent different areas of the same network, and the influences among the different databases are often not considered. In the construction of an economic network, however, more emphasis should be placed on considering the dependencies among different data sources and variables (*e.g.*, how stock market indices are influenced by exchange rates) than on patching together multiple and unrelated data sources.

Here, we introduce a new computational method that can integrate multiple economic variables, to produce a composite economic network. We used five monthly macro-economic variables–industrial production index (IPI), stock market index, consumer price index (CPI), exchange rate, and trade balance–for 18 countries, measured for a total of 192 months in the 1990s and 2000s. These five variables are generally used to describe the open economic model that accounts for cross-border trades. We included most of the G20 countries, as well as Spain and Portugal, in the analysis.

**Figure 1:**
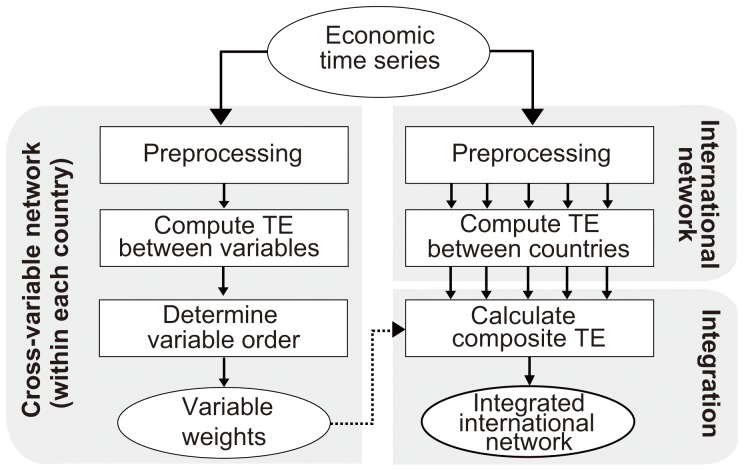
Overview of proposed approach. The proposed approach consists of three major steps – (1) cross-variable network construction within each country, (2) international network construction, and (3) integration by building a composite network based on the international and cross-variable networks.

Figure 1 shows an overview of the proposed approach, which consists of three major steps. First, we considered each country separately and measured the information transfer among the five variables within that country’s borders, thus yielding its domestic cross-variable network. Subsequently, we measured information transfer among different countries to build international networks. We measured the amount of information that transfers among the variables via transfer entropy (TE), which can measure directional information transfer by quantifying a deviation from conditional independence or a prediction improvement [19]. We tested the statistical significance of the measurement with a nonoverlapping block bootstrap method [20], [21]. Finally, we constructed a composite network based on the international networks and cross-variable networks created in the previous steps by using the proposed integrative approach.

## Results and Discussion

### Domestic Cross-variable Networks

We constructed a graphical representation called a *cross-variable network* in order to understand the information transfer between the five macro-economic variables in a given country. In this network, each node represents one macro-economic variable, and directed edges indicate the direction of information transfer between nodes. We measured the amount and direction of information transfer (or the degree of influence) by TE [19]. During the network construction, we retained only those edges whose TE values are statistically significant (*i.e.*, P<0.05), according to a statistical test based on nonoverlapping block bootstrapping [20], [21] (see Methods). Thus, not every pair of nodes in a cross-variable network has an edge. If there is an edge from node A (source) to node B (target), then we say that A *influences* B.

**Figure 2:**
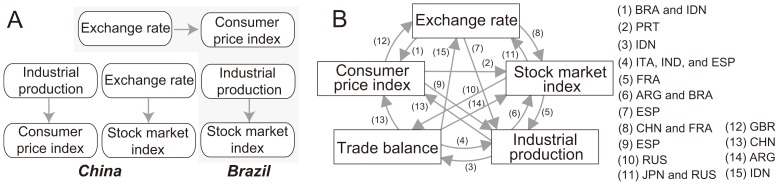
Domestic cross-variable networks. (A) Cross-variable networks for Brazil, and China, which are based on an 88-month time-series of the five variables and reveal the information transfer among the variables. (B) Cross-variable networks for all 18 countries in our study, superimposed in a single graph (the label of an edge indicates on which country’s cross-variable network the edge appears).

Figure 2A shows the cross-variable networks for Brazil and China, which are based on an 88-month time-series of the five variables and reveal the information transfer among the variables. For China, IPI affects CPI and exchange rate affects the stock market index. For Brazil, on the other hand, exchange rate influences CPI and IPI influences the stock market index. Figure 2B shows the cross-variable networks for all 18 countries in our study, overlaid in a single graph; the label of an edge indicates on which country’s cross-variable network the edge appears. According to the cross-variable networks in Figure 2B, various information transfer occurs among the five macro-economic variables, and the influence patterns are different for each country.

Figure 2 can also be interpreted from an economic viewpoint. One of the major topics of debate in macroeconomics is whether monetary variables such as inflation (CPI) and nominal exchange rate have causal effects on real variables including output (IP), investment (stock market index), export, and import (trade balance). Figure 2 shows that there is significant information transfer on both sides among real variables, and from nominal variables to real variables. Even though the causality chain cannot be confirmed without a relevant economic model, it is still interesting to see that monetary variables may be Granger causes [22] of real variables from our analysis. (In certain environments, non-zero transfer entropy and Granger causality are equivalent [23].).

### International Networks

**Figure 3:**
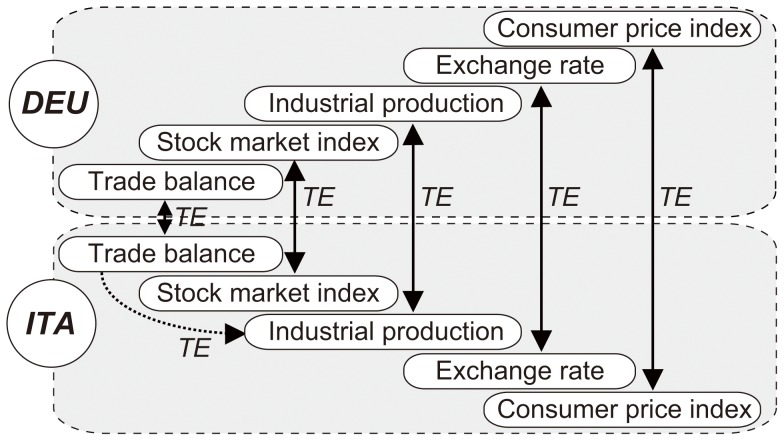
International network between Germany and Italy. This figure shows an international influence network between Germany and Italy (the cross-variable networks of the two countries are overlaid). A node represents a macro-economic variable, and a directed edge connects two nodes representing the same variable for two countries, if there is a statistically significant information transfer between the two nodes.

Using TE, we can also measure the information transfer among countries and construct an international influence network. Given two countries, we can determine the relationship between the same macro-economic variables for each of those two countries by calculating the transfer entropy between the two time-series. Figure 3 shows an international influence network between Germany and Italy, in which the cross-variable networks of the two countries are superimposed. In this type of network, a node represents a macro-economic variable, and a directed edge connects two nodes representing the same variable for two countries, if there is a statistically significant information transfer between the two nodes. Similar to a cross-variable network, we use nonoverlapping block bootstrapping to test the significance of an edge.

**Figure 4:**
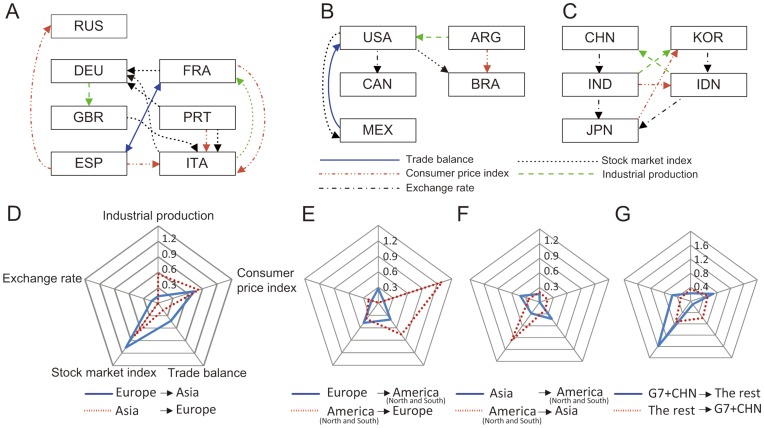
International networks. Figure 4A–C shows the international networks for three continents – (A) Europe, (B) North and South America, and (C) Asia. Figure 4D-F shows the outgoing TE values among the continents in terms of the five variables – (D) Asia and Europe, (E) the Americas and Europe, and (F) the Americas and Asia. Figure 4G shows the influence that the G7 countries (Canada, France, Germany, Italy, Japan, the UK, and the USA) and China have on the other countries in our study.

Figure 4A–C shows the international networks for three continents (*i.e.*, Europe, North and South America (“Americas”), and Asia), constructed by undertaking the above procedure. We show the information transfer for each of the five variables among the countries, by using different colors and line shapes (see the legend for Figure 4). For Europe, we did not include the exchange rate variable in the network, because the Euro currency appeared in 1999, which falls in the middle of our study’s data-collection period.

In Figure 4A–C, we can observe several defining features. First, more influence transfers among the countries in Europe than in Asia or the Americas. This indicates that economic variables are more closely bonded for European countries than countries in other areas, and this reflects the European countries’ cooperative movements, which led to the formation of the European Union. Second, in Figure 4A, the stock market index of Germany is a strong information receiver from other European countries, including France, Italy, and Portugal. In addition, the stock market index of Portugal acts as an information source for the indices of Germany (CDAX Composite), and Italy (FTSE). The CPI of Italy is a strong information receiver from other European countries.

We examine the influences of Italy, Portugal, and Spain, which have suffered from recent economic turmoil. In particular, Germany is the only common receiver of the significant information transfer from Italy and Portugal. Italy is a common receiver from France, Portugal, Spain and the UK. Given the strong tie among the European countries revealed by TE, we can expect that signs of the financial crisis originating from these countries will be transmitted, with either positive or negative annotation, to the rest of Europe. This transmission channel is somewhat obvious considering the role and importance of the German economy within the European Union. In addition, among the three countries, the influence of Spain on the other European countries is most noticeable. In terms of trade balance, France exchanges information with Spain; on the other hand, Italy and Russia receive information from Spain with respect to CPI.

We also observe some interesting traits in the Americas and Asia. The Americas have information transfer related to most of the five macro-economic variables used in the study. In particular, the USA acts as information sources of the stock market index (for Brazil and Mexico) and of exchange rate (for Canada) and information sinks of trade balance (for Mexico) and IPI (for Argentina). This finding points to the strong economic ties between the USA, the largest consumer in the Americas, and the other North American Free Trade Agreement countries and the two largest South American countries. In Asia, China, India and Japan form a chain of information transfer in terms of exchange rate, comparable to the relationship between South Korea, Indonesia and Japan. China and South Korea are information sinks of IPI for Indonesia and India, respectively. Japan influences South Korea in terms of CPI and acts as an information sink of exchange rate for India and Indonesia.

To determine how the three continent-areas (the Americas, Asia, and Europe) interact with each other, we measured the outgoing TE values among the continent-areas in terms of the five variables, as shown in Figures 4D (Asia and Europe), 4E (the Americas and Europe), and 4F (the Americas and Asia). For each variable, we accumulated its outgoing TE values for all of the countries in a continent-area and then normalized the sum by the number of countries. These radar charts reveal that different continent-areas have different influential variables. Table 1 lists the variables for the high outgoing TE values; these variables have normalized outgoing TE sums that are greater than the average for each continent-area. Based on our results, countries in the Americas and Europe have outgoing information transfer in the stock market index. European and North and South American countries influence each other in terms of different variables. None of the variables has an outgoing TE from Asia to either of the other two continent-areas, and this result is consistent with the findings of Kwon *et al*. [14].

**Table 1:** Information transfer among three continent-areas.

Direction	Variables of high (above-average) outgoing TE
Europe→Asia	Stock market index
Europe→Americas	None
Americas→Asia	Stock market index
Americas→Europe	Consumer price index
Asia→Europe	None
Asia→Americas	None

Figure 4G shows the influence that G7 countries (Canada, France, Germany, Italy, Japan, the UK, and the USA) and China have on the other countries in our study, in terms of the abovementioned five variables. In particular, the stock market index has outgoing TE from the G7 countries and China, to the other countries.

### Combining the Influence Transfer of Different Variables

To determine the economic interactions among the countries, we analyzed how the five macro-economic variables of one country collectively influence those variables in another country. For this approach, the information transfer of the five variables between the two given countries must be combined. A simple approach would be to sum the TE values of the five edges between the two countries in an international network, as shown in Figure 3. However, these variables typically have different levels of importance. Therefore, a sounder approach is to calculate the weighted sum of the five TE values. We determined the weight of a variable separately for the source and the target of an individual transfer, because its importance differs for each country. For example, we calculated the TE value for the composite information transfer from the USA to China, as follows:
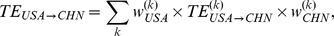
where 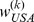
and 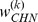 are the weights of the macro-economic variable *k* of the USA and China, respectively, and 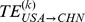 is the TE value of the information transfer for variable *k* from the USA to China. For a country, we determined the weights for the five variables based on their hierarchical order in the information transfer, as found in the country’s cross-variable network. This hierarchy appears on a cross-variable network as a maximum spanning tree (MST) [24]. The source of an information transfer is located at the root, and the sink nodes are at the leaves of the MST. The weight of the node is based on its proximity to the root (*i.e.*, the closer a node is to the root, the larger its weight). For example, in the cross-variable network of Brazil (Figure 2A), the IPI and exchange rate nodes have a higher order than the stock market index and consumer price index nodes, respectively. The Methods section provides additional details for determining the weight of each variable from a cross-variable network.

It should be noted that we chose a rather simple method in integrating international networks in the sense that each baseline network is constructed via a single macro-variable. Given the complex interactions of macro-economic variables across the borders, a natural extension of the proposed integration scheme may be used, for example,
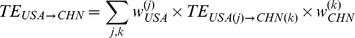
where 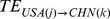 is the TE value of the information transfer from variable *j* of the USA to variable *k* of China. This extension is intuitive from our daily experience since we have seen that an interest rate cut in the USA affected not only the U.S. stock market, but also the interest rate in Japan. There are two reasons that we do not take this approach. First, the computational cost increases very fast as we move to a larger network. The computational cost rises at a quadratic rate in the number of variables if we consider all the international cross variable relations. It is significantly higher compared to a linear rate. Second, the current proposed method can easily handle the data set with multi-resolution or mixed frequency. Economic variables are observed in different frequencies. While the rate of inflation is officially announced every month in the most of countries, the stock market index can be observed every day or even every minute. Although the macro variables are carefully chosen and constructed to have the same monthly frequency in our current analysis, it is not actually required since the frequency matching in calculating the TE is not an issue as long as we use the same variables across countries. However, the extended integration scheme may cause a ‘small sample size’ problem if we want to include the growth rate of the gross domestic product (GDP) that is observed quarterly.

**Figure 5:**
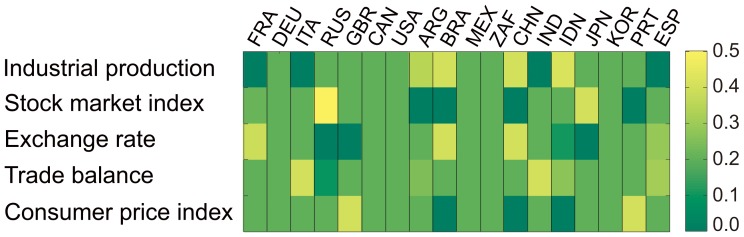
Variable weights. This figure shows the calculated weights for all the variables of the 18 studied countries. Each 5×1 column shows the color-coded weights for each country’s variables. This plot was drawn with the base entropy set to zero.

Figure 5 shows the calculated weights for all the variables of the 18 studied countries. Each 5×1 column shows the color-coded weights for each country’s variables. The trade balance is the most influential variable for India, Italy, and Spain, whereas the exchange rate is the most influential variable for Brazil, China, and France. The stock market index is the most influential for Russia and Japan. For six countries (Canada, Germany, Mexico, South Africa, South Korea, and the USA), no variable was found to bear a statistically significant influence.

### Adjusting the Degree of Impact by the Influence within the Border on the Composite Transfer Entropy

We utilized a parameter called the *base entropy* (BE), to adjust how much we consider the impact of the domestic influence transfer on the composite TE computation. Given a domestic cross-variable network, we determined the directions of edges in the associated MST as follows: We measured the TE between every two nodes and then left only those TE values that turned out to be statistically significant. For a pair of remaining nodes (*v*, *w*), we added edge *v*→*w* (*w*→*v*) to the MST if *TE_v→w_* (*TE_w→v_*) turned out to be statistically significant. In the MST constructed as above, we defined the BE as the TE of the node that has the lowest order (*i.e.*, the farthest node from the root). When the BE is zero, the domestic influence transfer affects the composite TE to the greatest extent. As the BE increases, the dependency of the composite TE on the domestic influence transfer decreases. Figure 5 was drawn with the BE set to zero.

**Figure 6:**
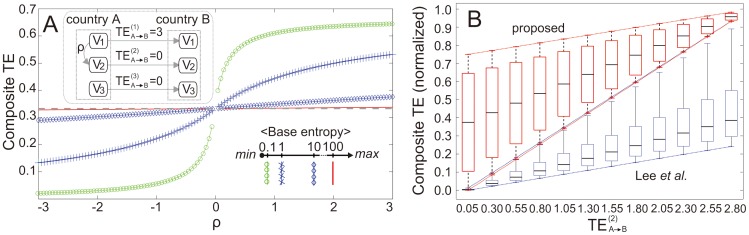
Analysis of composite transfer entropy calculation. (A) To examine the effect of adjusting the base entropy (BE) on the resulting composite TE value, this plot depicts how the composite 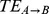 value varies as we change *ρ* with respect to four different BE levels (0.1, 1, 10, and 100). (B) Comparison between our approach and the integrative method proposed by Lee *et al*. [16], [17] in computational biology. This plot demonstrates how the composite TE value is affected by its component TE value.

To examine the effect of adjusting the BE on the resulting composite TE value, we performed a simulation study, as shown in Figure 6A. We assumed a two-country network, as drawn in the inset of the figure. Three macro-economic variables were considered, and we assumed that an influence transfer exists from 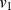 to 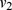, with a TE value of 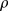. In the diagram, 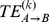 indicates the TE value of the information transfer between variable 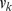 of the two countries. Figure 6A depicts how the composite 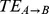 value varies as we change *ρ* with respect to four different BE levels (0.1, 1, 10, and 100). The dotted horizontal line on the plot represents the case in which we ignored the influence between 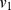 and 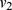 in calculating the composite TE. As we increase the BE level, the dependency of the composite TE on *ρ* deceases; the composite TE eventually becomes independent of *ρ* and converges to the dotted line. Figure 6B shows an interesting comparison between our approach and the integrative method proposed by Lee *et al*. [16], [17] in computational biology. This plot demonstrates how the composite TE value is affected by its component TE value. In this simulation, we used the same set-up shown in the inset of Figure 6A, except that we varied 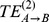 to examine its effect on the composite TE. We normalized the composite TE values from our method and that of Lee *et al*., due to there being differences in the signal ranges of the two methods. For a fixed value of 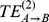, the range of the possible composite TE values is represented by a box plot (Figure 6B). The variability of the composite TE is introduced by using different levels for the BE in our approach, or an internal decaying parameter for the method by Lee *et al*. For both approaches, the median of the composite TE increases as 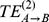 increases. For the proposed method, the variability of the composite TE decreases as 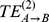 increases, and the opposite holds true for the method of Lee *et al*. In our approach, the variability (or uncertainty) of the composite TE decreases as its component TE increases, whereas Lee *et al*. designed their approach in the opposite way. Consequently, the composite TE can be nonzero in our approach, even when 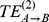 is zero. In contrast, the composite TE is zero if 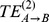 in the method by Lee *et al* is zero. Given that 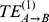 is nonzero, a nonzero composite TE may be more reasonable in this example.

The difference between the two methods originates from a difference in the basic principles of the application domain. In biological network integration, it seems reasonable to consider only those component TE values that are of a certain magnitude in computing the composite TE, in order to filter out noise. For the example shown in Figure 6A, the relationships between 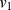 and 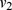 for biological network integration need not be considered, because they are independent variables obtained from separate databases. However, for the current problem, 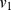 and 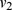 are not independent, and using an integration method for biological problems would yield incorrect composite TE values.

### Analysis of Composite Information Transfer

**Figure 7:**
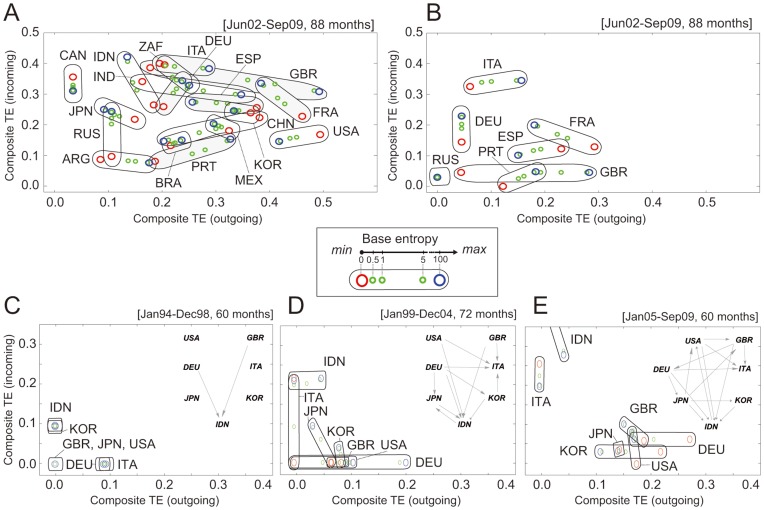
Composite information transfer. (A) The 18 countries are positioned according to their incoming and outgoing composite TE values. (B) The economic interactions among the European countries. Figure 7C–E shows the composite TE values for seven countries (Germany, Indonesia, Italy, Japan, South Korea, the UK, and the USA) over three periods – (C) January 1994-December 1998 (60 months), (D) January 1999-December 2004 (72 months), and (E) January 2005-September 2009 (60 months).

We integrated the influence transfer among the five macro-economic variables for the 18 countries. During the integration process, we determined two composite TE values (incoming and outgoing) for each country. In Figure 7A, the 18 countries are positioned according to their incoming and outgoing composite TE values. This plot is based on the time-series data collected from June 2002 to September 2009 (88 months). As previously explained, we swept the BE of a composite transfer from 0 to 100 (see the legend) to cover the different degrees of impact of the domestic influence transfer on the composite TE; thus, each country appears on the plot as a trajectory rather than a single point. A longer trajectory for a country reflects greater uncertainty in the composite TE value for that country (or the greater dependency of the composite TE on how much we consider domestic influence transfer).

From Figure 7A, we can cluster countries by their locations on the plot. The European countries and the USA have high outgoing composite TE values, confirming that these countries often serve as information sources in the global economy. In contrast, Argentina and Russia tend to have small outgoing and incoming composite TE values. We can also categorize countries according to trajectory length. Some countries, such as India, the UK and China, have long trajectories. It means that their composite TE values vary by the degree to which we consider domestic cross-variable information transfer with regard to the composite TE calculation. In other words, the domestic influence transfer of such countries can potentially affect their international influence transfer. Other countries, such as Brazil, Canada, Mexico, and South Korea, have short trajectories, suggesting that the domestic influence transfer of these countries does not substantially affect their international economic interactions. Another observation is the direction of the trajectories. Canada and Russia have vertical trajectories, but the locations of their minimum BE points are opposite of each other. For Canada, ignoring the impact of domestic influence transfer produces the largest amount of incoming composite TE, while the opposite is true for Russia.

Figure 7B shows that the economic interactions among the European countries are considerably strong. As only seven European countries are considered in the study (*i.e.*, France, Germany, Italy, Portugal, Russia, Spain, and the UK) for the composite TE calculation, the TE values of a country in Figure 7B differ from those of the same country in Figure 7A. The overall trajectory patterns of the European countries depicted in Figure 7B appear to be similar to those in Figure 7A. The incoming composite TE values for Germany are similar in both plots, but its outgoing composite TE level is much lower in Figure 7B. This suggests that Germany affects non-European countries more than its European neighbors do. In the case of Russia, the incoming and outgoing composite TEs are minute, which denotes that Russia has very weak connections to the other countries in Western Europe.

For some countries, the time series for four of the five macro-economic variables (except CPI) are available for a longer period (from January 1994 to September 2009, 192 months). To see the change in the trajectories of the composite TE values over time, we divided this period into three subperiods and calculated the composite TE values for seven countries (Germany, Indonesia, Italy, Japan, South Korea, the UK, and the USA) over each of these subperiods (Figure 7C–E). We can make several interesting observations from Figure 7C–E, whose insets show the international influence networks constructed with composite TE values.

The amount of composite TE of Germany has gradually increased over the three subperiods. Reunification of Germany occurred in 1990, only a few years prior to the start of the first subperiod (Figure 7C). We conjecture that the German government put considerable effort into handling the domestic aftermath of reunification during the initial period (Figure 7C) and that the international influence of the German economy was reduced in the first subperiod, although a certain amount of outgoing influence is still observable in Figure 7C. In the second subperiod (Figure 7D), the incoming composite TE of Germany remains negligible, but the outgoing composite TE starts to increase with a somewhat long trajectory. During this subperiod the economic integration among EU countries became a strong bond. Especially in 1999 a monetary union, Eurozone, was established in which a common monetary unit started to be circulated. The German economy has played an important role in the establishment of the Eurozone, and this increased influence is captured in the outgoing TE. In the last subperiod (Figure 7E), the outgoing composite TE of Germany becomes larger with shorter trajectory than in the second subperiod. The incoming composite TE is also greater. This observation suggests that Germany becomes close to completion of its reunification process in the last subperiod and expands its influence in European economy.

The changes in the incoming composite TE values of Indonesia and Italy appear more salient than Germany. For Italy, its outgoing composite TE tends to decrease over these three periods, but its incoming composite TE shows an increasing pattern. It would be interesting to investigate if this observation bears any relationship with the European sovereign debt crisis that certain European countries recently faced. For Indonesia, its outgoing composite TE does not change considerably over the three periods, but its incoming composite TE grows fast. In the late 1990s, Indonesia experienced a severe financial crisis associated with foreign exchange [25], after which the Indonesian Rupiah has never been recovered its previous high valuation. The political instability after the crisis also may result in this rapid increase of the incoming composite TE.

South Korea, which also suffered from the same Asian financial crisis in 1997 as Indonesia, shows different changes in the composite TE. In the first subperiod (Figure 7C), the outgoing composite TE of South Korea is negligible, whereas the incoming composite TE is more noticeable. As a developing country at that time, South Korea continued to accept foreign investments and aid to rebuild the country, but its influence to other countries was insignificant. During the first subperiod, South Korea even faced the financial crisis as mentioned above. However, this crisis eventually provided an opportunity for South Korea to make its export-driven economy stronger than before. South Korea is now a member of the Organization for Economic Co-operation and Development (OECD) and has a strong economy driven by exports and foreign trades. This fact may be reflected in Figure 7D and 7E, where the outgoing composite TE of South Korea tends to increase.

In Figure 7D–E, the incoming composite TE of Japan seems to decrease. After the Asian currency crisis in 1997 [25], Japanese banks, which already weakened from the long recession, suffered capital losses as the crisis deepened and had to collect back their outstanding international loans to other Asian countries to meet the capital adequacy requirement. This may appear in Figure 7D as the incoming composite TE of Japan at certain levels. In the last subperiod, the trajectory of the composite TE of Japan appears as a single point, meaning that changing the degree of domestic cross-variable influence on international influence transfer makes little difference. Many factors may be responsible for this phenomenon. One explanation is that the international economic interaction of Japan started to shrink with the burst of the domestic real estate bubble that resulted in a several decade-long stagnation. This shrinkage took place mainly because the focus of the government’s main economic policy had moved from the trade related issues to the domestic debt related ones. Even so, Japanese economy is expected to maintain its influence on the global economy since it has constantly shown massive trade surpluses against the rest of the world [26]–[28]. This expectation may be reflected on the moderate growth of the outgoing composite TE values in Figure 7D–E.

Note that Figure 7C–E was obtained from the integrated TE values rather than individual TE calculations. As for the integrated TE analysis, it would deserve new research to uncover the effect of (non)stationarity of individual component TE values underlying the composite TE computation. In the preprocessing of our methodology, we carried out first-differencing and discretization, which are effective in reducing nonstationarity, especially for unit-root time series.

## Methods

### Data Preparation

Our study focused on 18 countries. Their names and three-letter abbreviations as denoted by ISO3166-1 alpha-3 code [29] are as follows. *Europe*: France (FRA), Germany (DEU), Italy (ITA), Portugal (PRT), Russia (RUS), Spain (ESP), and the United Kingdom (GBR); *North and South America*: Argentina (ARG), Brazil (BRA), Canada (CAN), Mexico (MEX), and the United States (USA); *Asia*: China (CHN), India (IND), Indonesia (IDN), Japan (JPN), and the Republic of Korea (KOR); *Africa*: South Africa (ZAF). These countries include most of the G20 countries. Among the G20 countries, we excluded Turkey, Saudi Arabia, and Australia, due to lack of data.

We used five monthly macro-economic variables: IPI, exchange rate (per special drawing rights; see [30]), stock market index, trade balance, and CPI. We obtained datasets from the CEIC Macro-economic Databases for emerging and developed markets [31]. CPI data are from June 2002 to September 2009 (88 months); the other variables are from January 1994 to September 2009 (192 months). China’s IPI data had four missing data-points (January of 2009, 2008, 2007, and 2006); we performed interpolation to estimate them. We used seasonally adjusted time-series data for IP and trade balance.

For stationarity, we first-differenced the variables. To calculate the TE, we discretized the first-differenced series to integer values, according to the following procedure. We set three threshold points (at the mean and two points that are one standard deviation away from the mean) that define four states: fast increasing, increasing, decreasing, and fast decreasing. We coded these four states, from fast increasing to fast decreasing, using the integers 1, 2, 3, and 4. In the discretization by binning, using too few bins may reverse the detected information transfer in comparison to the true information transfer [32]. In our experiments, using 4 and 6 bins produced similar results, whereas using 2 bins reversed approximately half of transfer directions with respect to the 4-bin case.

### Transfer Entropy

We employed TE [19] to measure information transfer. Transfer entropy was proposed to measure information transfer between two time-series data based on the probability density function. In contrast to mutual information and correlation, TE can analyze the directions of each information transfer.

For a more formal definition of TE, let 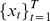 be a time series that follows a stationary Markov process with order *p*, that is, 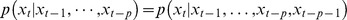. For notational convenience, we denote 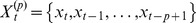. Consider another time series 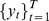. If the generalized Markov property holds, *i.e.*,
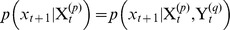
the knowledge of the past realization of 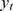 does not improve the prediction of 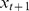. When the prediction is carried out in a linear regression setting with 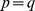, the generalized Markov property implies Granger non-causality. For simplicity of notation, let 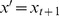, 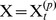, and 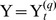. We define the transfer entropy from 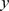 to 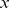 as the expected value on the conditional Kullback-Leibler divergence that measures the violation of the generalized Markov property:



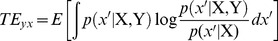
which we can rewrite as



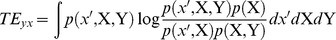



For an implementation of TE, we can consider the sample counterpart
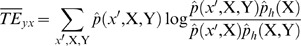
where 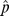 denotes the density estimated with time series 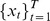 and 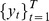. For example, we can apply a kernel estimator (with bandwidth *h*)



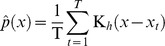



Under regularity conditions, we can show the convergence of 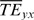 to 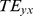 as T and *h* approach to zero. In our current implementation, we follow the standard practice in the literature where the relative frequency (with coded data as described in the previous section) is applied to get the estimated density, 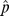.

Our data consist of time series of scalar variables, but the Markov processes TE is defined on are defined in a vector state space. We thus employed the Cao criterion [33] and the Ragwitz criterion [34] to check if our data need the method of time-delay embedding [33], [34]. It can reveal the evolution of the vector field underlying a scalar observation. (For implementation, we used the TRENTOOL package [38].) Based on the result of this check, we performed time-delay embedding with the dimension of two on our time-series data prior to calculating TE.

### Testing Statistical Significance of Transfer Entropy by Bootstrapping

After measuring the TE from the time-series J to I, we applied the nonoverlapping block bootstrap method [20], [21] to test the statistical significance of the measured TE. We first divided each of the two time-series randomly into two blocks at a random cut point and formed a new time-series by rejoining the blocks with bootstrap sampling [20], [21]. Using too many small non-overlapping blocks in the bootstrap will destroy any nuisance nonstationarity remaining in the data and thereby bias the bootstrap towards a false positive result. We then measured the TEs from J to I. Replicating this process 1,000 times produces a TE distribution for the two time-series, from which we can test the statistical significance of a specific TE value. The significance level is set at 0.05. There is also an issue of multiple comparison given that we test the significance of 1890 TE values in total [306 (inter-country) times 5 (economic variables) plus 20 (domestic) times 18 (countries)]. We performed the multiple comparison correction based on the false discovery rate (FDR) [35]–[37] with the FDR threshold of 0.05. In the end, 119 TE values turned out to be statistically significant out of the 1890 TE values (approximately 6.5%).

**Figure 8:**
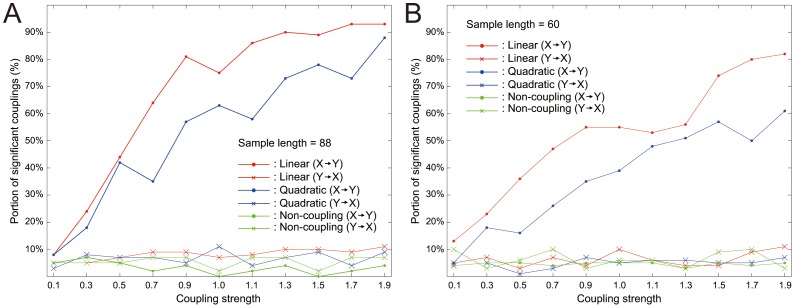
Validity check for testing statistical significance of transfer entropy by non-overlapping block bootstrapping. Portion of statistically significant couplings over coupling strengths from 0.1 to 1.9. Order n = 2. Three types of coupling (linear, quadratic, none) used. Assumed direction of information transfer: X→Y. Data lengths: 88 (A) and 60 (B).

For further verification of this bootstrapping-based testing, we utilized the synthetic autoregressive process with order n, according to Vincente *et al*. [39]:
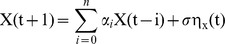


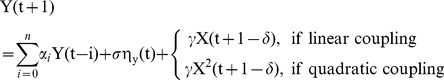
where 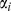 is a parameter drawn from a normalized Gaussian distribution, 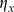 and 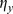 are Gaussian white noise, and three parameters 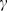, 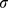, and 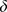 represent the coupling strength, noise strength and delay, respectively. Using this model, we generated signals X and Y, assuming information transfer from X to Y. We then measured the TE values and computed p-values using the non-overlapping block bootstrap method as described above. We repeated the procedure for 100 different combinations of X and Y. Figures 8A and 8B show the fraction of statistically significant TE values (*P*<0.05) over different coupling strength values for data sets with lengths 88 and 60, respectively. The coupling strength was set to the ratio of the first two terms to the last term in the above equation for Y(t+1). The fraction of significant couplings increases under the linear and quadratic coupling conditions for X→Y, whereas there is no notable change for the non-coupling case and TE values of Y→X. This result suggests that the non-overlapping block bootstrap method works for the purpose of statistical significance testing in this study. See Figure S1 in Supplementary Material S1 for more details.

### Updating Variable Weights for Computing Composite Transfer Entropy

We integrated the individual information transfer appearing in the domestic cross-variable networks, under the following assumptions. First, the composite information transfer between two countries is a linear combination of individual component transfer. Second, the weight of an influential variable (or an information source) is higher than that of an influenced variable (or an information sink).

For each country, the variables are initially equally weighted one-fifth each, and we updated the variable weights based on the influence transfer between the variables represented in the MST [24] of the country’s cross-variable network. MST is useful, because we can find the direction of the overall influence transfer among the variables therefrom. For example, the USA has only an information transfer from the exchange rate to the stock market index, and no other transfer (Figure 2). We initially set the weights of the five variables as follows:
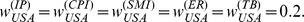



Then, the weights of the exchange rate and the stock market index variables become
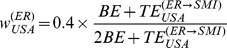
and



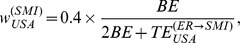
where *BE* represents the base entropy.

## Supporting Information

Supplementary Material S1 **Figure S1, Measuring transfer entropy over various coupling strengths.** Each TE computation was repeated 1000 times and shown are the average values. Data lengths = 88 and 60. Order n = 1, 2, 3, and 4. Three types of coupling (linear, quadratic, none) used. Assumed direction of information transfer: X→Y.
(PDF)
