# Supplementary material for: Entropy-Based Analysis and Bioinformatics-Inspired Integration of Global Economic Information Transfer
Source: PLoS One. 2013 Jan 2;8(1):e51986. doi: 10.1371/journal.pone.0051986 (PMC3534713; doi:10.1371/journal.pone.0051986)
Supplement: Supplementary Material S1 — Figure S1, Measuring transfer entropy over various coupling strengths. Each TE computation was repeated 1000 times and shown are the average values. Data lengths = 88 and 60. Order n = 1, 2, 3, and 4. Three types of coupling (linear, quadratic, none) used. Assumed direction of information transfer: X→Y. (PDF) [file pone.0051986.s001.pdf]

[Supplementary Material]

Entropy-based analysis and bioinformatics-inspired integration of global economic information transfer

J. Kim, G. Kim, S. An, Y.-K. Kwon, and S. Yoon

In this supplementary material, we address the following issues:

1. Does it make sense to compute TE for data sets of length 60 or 88?
2. Does the non-overlapping block bootstrap method work for the purpose of testing statistical significance of TE values?

Setup

Let us consider the synthetic AR(n), an autoregressive process with order n:

$$\mathrm{X}(\mathrm{t}+1)=\sum_{i=0}^{n}\alpha_i X(t-i)+\sigma\eta_x(t)$$

$$\mathrm{Y}(\mathrm{t}+1)=\sum_{i=0}^{n}\alpha_i Y(t-i)+\sigma\eta_y(t)+\begin{cases}\gamma X(t+1-\delta), & \text{if linear}\\ \gamma X^2(t+1-\delta), & \text{if quadratic}\end{cases}$$

where α_i is a parameter drawn from a normalized Gaussian distribution, η_x and η_y are Gaussian white noise, and three parameters γ, σ and δ represent the coupling strength, noise strength and delay, respectively.

Based on the above, we experimented for $\mathrm{n}=1,2,3,4$ under linear, quadratic and no coupling conditions in order to address the two issues. The lengths of the data sets used were 60 and 88. (Note that these are the lengths of the data sets used in the main article.) We set $\sigma=1$ and $\delta=0$.

Issue 1: Does it make sense to compute TE for data sets of length 60 or 88?

Figure S1 shows the computed TE values over various coupling strengths from 0.1 to 1.9 for the three different coupling conditions. In this experiment, we assume that information transfers from X to Y. We also assume the degree of information transfer is represented by the coupling strength: the higher coupling strength, the more information transfer. That is, from the formula for $Y(t+1)$

$$\mathrm{Y}(\mathrm{t}+1) = \underbrace{\sum_{i=0}^{n} \alpha_i Y(t-i) + \sigma\eta_y(t)}_{\text{Signal term}} + \underbrace{\begin{cases} \gamma X(t+1-\delta), & \text{if linear} \\ \gamma X^2(t+1-\delta), & \text{if quadratic} \end{cases}}_{\text{Coupling term}}$$

we defined the coupling strength as the ratio of the variance of the coupling term to the variance of the signal term, namely

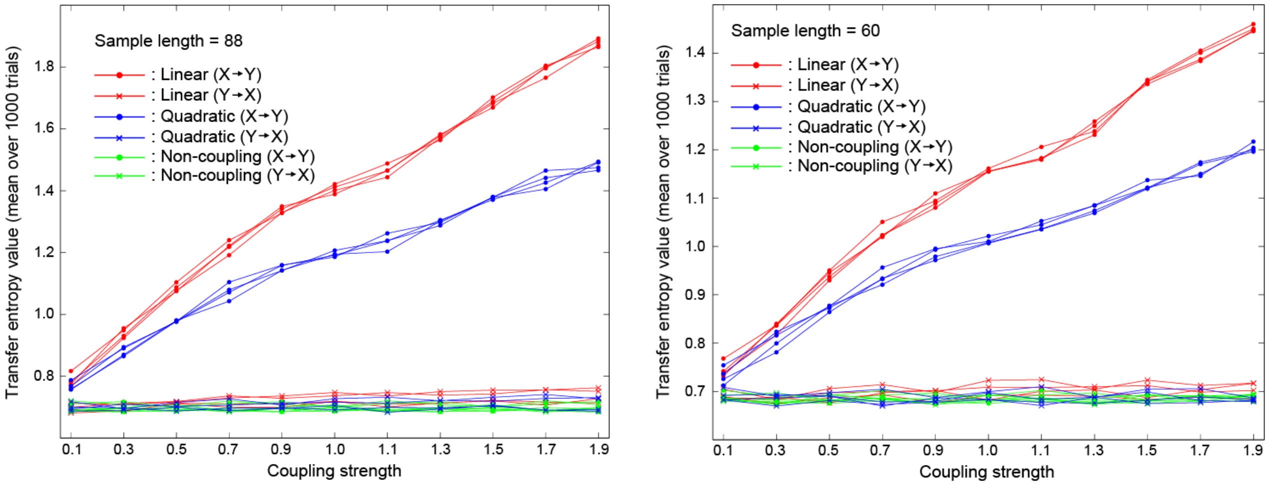

Figure S1: Measuring transfer entropy over various coupling strengths. Each TE computation was repeated 1000 times and shown are average values. Data lengths = 88 and 60. Order $n = 1, 2, 3$, and 4. Three types of coupling (linear, quadratic, none) used. Assumed direction of information transfer: $X \rightarrow Y$.

$$\text{Coupling strength} = \frac{var\ (\text{coupling term})}{var\ (\text{signal term})}$$

In Figure S1, we can observe that the order n does not affect the overall outcome. As we assumed, we can see that the information transfer from X to Y appears as higher values of TE than that of the opposite direction. When no coupling is used, the TE values were negligible over the

entire range of coupling strength. Taken together, this set of experiments demonstrates that TE value computation makes sense for data sets of lengths 60 and 88.

Issue 2: Does the non-overlapping block bootstrap method work for the purpose of testing statistical significance of TE values?

Based on the AR(n) setup, we carried out additional experiments. This is to see the validity of the statistical significance testing based on non-overlapping block bootstrapping, as described in the main article. After generating signals X and Y using the AR(2) model (there was little dependency on the choice of n), we measured the TE values and computed p-values using the non-overlapping block bootstrap method (2 blocks using random cuts; repeated 100 times). We left only those TE values whose p-values are less than 0.05. We repeated the above procedure for 100 different combinations of X and Y.

Figure 8 in the main article shows the fraction of statistically significant TE values over different coupling strength values for data sets with lengths 88 and 60. As shown in Figure 8, the fraction of significant couplings increases under the linear and quadratic coupling conditions for $X \rightarrow Y$, whereas there is no notable change for the non-coupling case and TE values of $Y \rightarrow X$. This result suggests that the non-overlapping block bootstrap method works for the purpose of statistical significance testing in the current context.
